# Somatic mutations in benign breast disease tissue and risk of subsequent invasive breast cancer

**DOI:** 10.1038/s41416-018-0089-7

**Published:** 2018-06-06

**Authors:** Thomas E. Rohan, Christopher A. Miller, Tiandao Li, Yihong Wang, Olivier Loudig, Mindy Ginsberg, Andrew Glass, Elaine Mardis

**Affiliations:** 1 Department of Epidemiology and Population Health, Albert Einstein College of Medicine, 1300 Morris Park Avenue, Bronx, NY 10461 USA; 2 McDonnell Genome Institute, Washington University School of Medicine, St. Louis, MO USA; 3 Department of Pathology and Laboratory Medicine, Rhode Island Hospital and Lifespan Medical Center, Warren Alpert Medical School of Brown University, Providence, RI USA; 4 Hackensack University Medical Center, David Joseph Jurist Research Center, Hackensack, NJ USA; 5 Center for Health Research, Kaiser Permanente Northwest, Portland, OR USA; 6 Institute for Genomic Medicine, Nationwide Children’s Hospital and The Ohio State University College of Medicine, Columbus, OH USA

**Keywords:** Cancer epidemiology, Risk factors

## Abstract

**Background:**

Insights into the molecular pathogenesis of breast cancer might come from molecular analysis of tissue from early stages of the disease.

**Methods:**

We conducted a case–control study nested in a cohort of women who had biopsy-confirmed benign breast disease (BBD) diagnosed between 1971 and 2006 at Kaiser Permanente Northwest and who were followed to mid-2015 to ascertain subsequent invasive breast cancer (IBC); cases (*n* = 218) were women with BBD who developed subsequent IBC and controls, individually matched (1:1) to cases, were women with BBD who did not develop IBC in the same follow-up interval as that for the corresponding case. Targeted sequence capture and sequencing were performed for 83 genes of importance in breast cancer.

**Results:**

There were no significant case–control differences in mutation burden overall, for non-silent mutations, for individual genes, or with respect either to the nature of the gene mutations or to mutational enrichment at the pathway level. For seven subjects with DNA from the BBD and ipsilateral IBC, virtually no mutations were shared.

**Conclusions:**

This study, the first to use a targeted multi-gene sequencing approach on early breast cancer precursor lesions to investigate the genomic basis of the disease, showed that somatic mutations detected in BBD tissue were not associated with breast cancer risk.

## Introduction

One model of the natural history of breast cancer posits that it develops as a result of the progression of breast tissue through specific histological forms of benign breast disease (BBD) and then carcinoma in situ before ultimately developing into invasive breast cancer (IBC)^1^. Consistent with this, women with a history of BBD have a two-fold increase in the risk of developing subsequent IBC^1^.

Predicting the behavior of BBD requires an understanding of its underlying biology^2^. In this regard, insights into the molecular pathogenesis of breast cancer will potentially come from analyses conducted on tissue from early stages of the disease^2,3^. Almost inevitably, for studies attempting to relate early molecular changes to the likelihood of subsequent invasive cancer, this necessitates the use of formalin-fixed, paraffin-embedded (FFPE) archival tissue, because it obviates the need for both prospective collection of data and tissue and for subsequent long-term follow-up to ascertain outcome.

In the prospective study reported here, we examined the association between somatic mutations detected in BBD tissue and risk of subsequent IBC.

## Materials and methods

### Study population

The study population has been described in detail elsewhere^4^. In brief, the study was conducted in a cohort of 15,395 women who had biopsy-confirmed BBD diagnosed between 1971 and 2006 at Kaiser Permanente Northwest (KPNW). Subsequent IBC occurrence (to mid-2015) was ascertained by linking records from the BBD cohort to the KPNW Tumor Registry. Institutional Review Board approval was obtained at all participating sites, and because the data/specimens were not collected specifically for this research project and did not contain a code derived from individual personal information, the study was considered not to meet the definition of human subject research as defined by 45 CFR 46, 102(f).

### Study design/sample size

We conducted a case–control study nested within the BBD cohort. Cases were women with BBD who subsequently developed IBC. Using risk-set sampling, one control was selected for each case and was matched to the corresponding case on age at diagnosis of BBD (+/−1 year) (and implicitly, given the risk-set sampling, on duration of follow-up); controls were sampled randomly from the risk-sets with replacement. In addition to being alive and free of IBC, each control was required not to have undergone a mastectomy before the date of diagnosis of breast cancer for its matched case. The study was restricted to those who had adequate quantity and quality of DNA extracted from both the lesion and from the adjacent normal tissue (see below) and successful sequence generation. This led to the exclusion of 13 samples, leaving 218 case–control pairs.

### Histopathology/clinical data

FFPE blocks of BBD tissue were retrieved from storage. Haematoxylin and eosin-stained sections were prepared and were reviewed and classified according to standard histological criteria^1,5,6^. Specifically, the BBD lesions were classified into the following categories: (1) nonproliferative disease, (2) proliferative disease without atypia, and (3) proliferative disease with atypia (atypical ductal hyperplasia, atypical lobular hyperplasia, or both). Specimens were designated as having proliferative changes if they contained any of the following: ductal hyperplasia, papilloma, radial scar, or sclerosing adenosis. Cysts, aopcrine metaplasia, fibroadenoma without epithelial hyperplasia, or columnar cell change were considered to be non-proliferative unless they contained one of the listed proliferative lesions. Columnar cell lesions and flat epithelial atypia were also evaluated based on the World Health Organization criteria^6^: columnar cell change and hyperplasia were categorised as proliferative disease without atypia, and flat epithelial atypia was categorised as proliferative disease with atypia. Data on clinical/epidemiologic factors were extracted from medical records.

### Targeted sequence capture and sequencing

DNA was extracted separately from the BBD lesions and from adjacent normal tissue (the latter enabling putative germline variants to be excluded). Sequencing libraries were made from samples with as little as 8.1 ng of input DNA, although the mean input amount was 70.1 ng. An 83-gene panel was designed to target all the exons of genes (see Supplementary Table 1) that were selected based on their known importance in breast cancer, as demonstrated by the The Cancer Genome Atlas breast cancer study and others. The use of this targeted sequence capture approach and the sequencing were performed as described previously^7^.

**Table 1:** Gene list for targeted sequencing

KRAS	MYB	NF1	SF3B1	AKT1	CBFB`
CTCF	FOXA1	MDM2	MDM4	MLL2	MLL3
NCOR1	PIK3R1	PTEN	TBX3	AGTR2	ATR
BIRC6	BRAF	CDH1	CDKN1B	CSF1R	DDR1
ERBB2	GATA3	INSRR	JAK1	JAK2	KIT
LTK	LYN	MALAT1	MAP2K4	MAP3K1	MET
PDGFRA	PIK3CA	RB1	RUNX1	TP53	ESR1
SMG1	ERBB3	ERBB4	MTOR	FRG1B	MAP3K4
MLL	ATM	NOTCH4	PRKDC	PRLR	RELN
BRCA1	BRCA2	NCOA3	NCOR2	ARID1A	MED12
AKT2	AKT3	ARID1B	AURKA	CASP8	CAV1
FBXW7	FOXC1	FZD7	MAGI3	MAP3K13	MTAP
MYBL2	PIN1	PPP2R2A	RB1CC1	RERG	SMARCD1
TAB1	TAB2	TGFB1	TGFB2	XBP1	

See Chanock et al^9^. ; Banerji et al^8^. ; Curtis et al^10^. ; and Stephens et al ^11^.

### Data analysis

Somatic single-nucleotide variants (SNVs) and short indels were detected using the Genome Modeling system^8^. Sequence data were aligned to reference sequence build GRCh37-lite-build37 using bwa version 0.5.9^9^ (parameters: −*t* = 4, −*q* = 5), then merged and deduplicated using picard version 1.46. SNVs were detected using the union of four callers: (1) samtools version r982^10^ (params: mpileup -BuDs) intersected with Somatic Sniper^10^ version 1.0.2 (params: -F vcf -q 1 -Q 15) and processed through false-positive filter v1 (params: --bam-readcount- version 0.4 --bam-readcount-min-base-quality 15 --min-mapping-quality 40 --min-somatic-score 40), (2) VarScan^11^ version 2.3.6 filtered by varscan-high-confidence filter version v1 and processed through false-positive filter v1 (params: --bam-readcount-version 0.4 --bam-readcount-min-base-quality 15), (3) Strelka^11^ version 1.0.11 (params: isSkipDepthFilters = 0), and (4) Mutect version 1.1.4. Indels were detected using the union of three callers: (1) GATK somatic-indel version 5336^12^, (2) VarScan version 2.3.6 filtered by varscan-high-confidence- indel version v1, and (3) Strelka version 1.0.10 (params: isSkipDepthFilters = 0).

SNVs and indels were further filtered by requiring 20× coverage, removing artifacts found in a panel of 905 normal exomes, removing sites that exceeded 0.1% frequency in the 1000 genomes or NHLBI exome sequencing projects, and then using a Bayesian classifier (https://github.com/genome/genome/blob/master/lib/perl/Genome/Model/Tools/Validation/IdentifyOutliers.pm) and retaining variants classified as somatic with a binomial log-likelihood of at least 5.

Samples were screened for FFPE artifacts by first identifying mutations with appropriate dinucleotide mutation context (CG > TG) ref: https://www.ncbi.nlm.nih.gov/pmc/articles/PMC4912568/ and variant allele frequency (VAF) <10%. Eighteen samples were identified with at least three such putative artifacts, suggesting that these samples had been adversely affected by damage due to formalin fixation. Eighty four mutations flagged as artifacts in these samples were removed from further consideration.

Copy number variant calling was attempted, but the density of the probes in this targeted panel was insufficient to enable accurate inference.

All statistical tests were performed with R version 3.3.1.

## Results

**Fig. 1:**
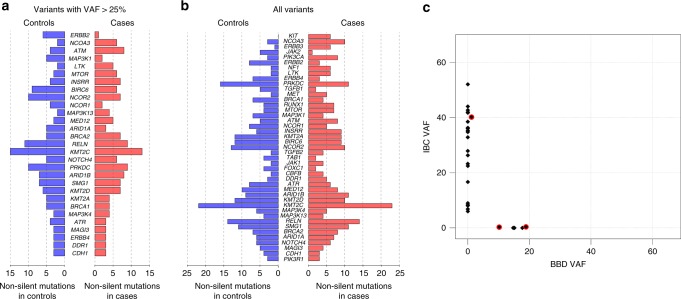
**a**, **b** Number of cases (right) and controls (left) with non-silent mutations in specific genes. Shown are all genes where at least five cases had mutations. Genes were tested for significant differences between cases and controls using paired *t*-test and are ordered by *p*-value—none reached significance after multiple testing correction. **c** Variant allele frequencies (VAFs) of all mutations found in paired BBD and IBC samples from seven patients. Red highlights indicate mutations with non-zero VAFs in both samples (all had two or fewer supporting reads in one of the samples)

We sequenced the protein-coding exons of 83 genes in DNA extracted from tissue samples from 436 patients (218 pairs of matched case/control BBD samples, as well as 218 pairs of matched normal tissue samples). We detected 504 somatic mutations in the cases and 497 in the controls (mean variant coverage 90.4×) with no significant difference in overall mutation burden (via paired *t*-test, Supplementary Table 2a). Restricting the comparison to non-silent mutations gave counts of 332 mutations in the cases and 333 in the controls. No individual gene had significantly different numbers of mutations between the cases and controls, whether considering all mutations or only non-silent mutations (Fig. 1a). This was true whether considering putative founding clone mutations (VAF > 25%) or all mutations (Fig. 1b). One gene, *KIT*, was exclusively mutated in patients who progressed to IBC but failed to reach statistical significance after multiple testing correction (paired *t*-test, *p* = 0.0302, False Discovery Rate = 1). No substantial differences between cases and controls were observed in the nature of mutations within genes (i.e., *PIK3CA*^(1047)^ vs other *PIK3CA* mutations). We also examined mutational enrichment at the pathway level, using ConsensusPathDB^13^ and, alternatively, by taking the nearest neighbors of each gene in protein–protein interaction networks obtained from Genemania^14^. No significant pathway enrichment was observed.

For seven subjects, we obtained tissue samples from the subsequent ipsilateral IBC. We sequenced DNA from these samples using the same targeted panel of genes described above. In total, 28 mutations were observed, and none was shown definitively to be shared between the BBD and IBC (Fig. 1b, Supplementary Table 2b).

## Discussion

This is the first study that has used a targeted multi-gene sequencing approach on early breast cancer precursor lesions to investigate the genomic basis of the disease. Though not statistically significant, the exclusivity of KIT mutations to lesions that progressed to IBC is nonetheless deserving of further investigation in a larger cohort. Overall, the null results may reflect sample size limitations, the limited gene set and regions analysed, and misclassification of mutation status due to impaired DNA quality. The fact that somatic mutations were observed to be private between the BBD and IBC samples likely arises from the fact that the BBD biopsies were both spatially and temporally distinct from the IBC biopsies. In each case, we clearly did not sample the population of cells that ultimately gave rise to the tumour. Without a more comprehensive assay (that includes all mutations and copy number alterations), we cannot say whether the BBDs were completely independent clonal expansions or whether they shared key founding mutations that we did not detect (perhaps copy number events, which are frequently observed as “early” events in tumour evolution). In the latter case, the BBD biopsies would represent a “dead end” tumour subclone that was ultimately outcompeted by other tumour cells with additional mutations and increased fitness.

Despite the null results reported here, further investigation, exploiting the vast archives of FFPE breast tumour tissue with clinical outcome data using similar or even more detailed approaches (e.g., exome/whole-genome sequencing) to those employed here, is warranted. Such work has translational potential given that identification of DNA changes associated with increased risk may allow early detection of women at risk for breast cancer and may foster the development of new approaches to the clinical management of women with BBD^2,15^.

## Electronic supplementary material

Supplementary Table 1

Supplementary Table 2
