# Supplementary material for: Somatic mutations in benign breast disease tissue and risk of subsequent invasive breast cancer
Source: Br J Cancer. 2018 Jun 6;118(12):1662–4. doi: 10.1038/s41416-018-0089-7 (PMC6008400; doi:10.1038/s41416-018-0089-7)
Supplement: Supplementary file 1 — Supplementary Table 1 [file 41416_2018_89_MOESM1_ESM.docx]

**Supplementary Table 1. Gene list for targeted sequencing.^a^**

| KRAS | MYB | NF1 | SF3B1 | AKT1 | CBFB |
| --- | --- | --- | --- | --- | --- |
| CTCF | FOXA1 | MDM2 | MDM4 | MLL2 | MLL3 |
| NCOR1 | PIK3R1 | PTEN | TBX3 | AGTR2 | ATR |
| BIRC6 | BRAF | CDH1 | CDKN1B | CSF1R | DDR1 |
| ERBB2 | GATA3 | INSRR | JAK1 | JAK2 | KIT |
| LTK | LYN | MALAT1 | MAP2K4 | MAP3K1 | MET |
| PDGFRA | PIK3CA | RB1 | RUNX1 | TP53 | ESR1 |
| SMG1 | ERBB3 | ERBB4 | MTOR | FRG1B | MAP3K4 |
| MLL | ATM | NOTCH4 | PRKDC | PRLR | RELN |
| BRCA1 | BRCA2 | NCOA3 | NCOR2 | ARID1A | MED12 |
| AKT2 | AKT3 | ARID1B | AURKA | CASP8 | CAV1 |
| FBXW7 | FOXC1 | FZD7 | MAGI3 | MAP3K13 | MTAP |
| MYBL2 | PIN1 | PPP2R2A | RB1CC1 | RERG | SMARCD1 |
| TAB1 | TAB2 | TGFB1 | TGFB2 | XBP1 |  |

^a^ See Banerji, S. et al. Sequence analysis of mutations and translocations across breast cancer subtypes. *Nature* 486: 405-409 (2012); Chanock, S.J. et al. Somatic sequence alterations in twenty-one genes selected by expression profile analysis of breast carcinomas. *Breast Cancer Res*. 2007;9:R5 (2007); Curtis, C. et al. The genomic and transcriptomic architecture of 2,000 breast tumours reveals novel subgroups. *Nature* 486: 346-352 (2012); Stephens, P.J. et al. The landscape of cancer genes and mutational processes in breast cancer. *Nature* 486: 400-404 (2012).

**Supplementary Table 2a.** Mutations identified in 436 BBD samples (218 case-control pairs).

**Supplementary Table 2b.** Mutations identified in 7 paired BBD and IBC samples.
